# Can a bank crisis break your heart?

**DOI:** 10.1186/1744-8603-4-1

**Published:** 2008-01-15

**Authors:** David Stuckler, Christopher M Meissner, Lawrence P King

**Affiliations:** 1 University of Cambridge, Faculty of Social & Political Sciences, Free School Lane, Cambridge CB2 3RQ, UK; 2 Faculty of Economics, University of Cambridge and National Bureau of Economic Research, Cambridge, CB3 9DD, UK; 3 Department of Economics, University of California, Davis, One Shields Avenue, Davis, CA 95616, USA

## Abstract

**Background:**

To assess whether a banking system crisis increases short-term population cardiovascular mortality rates.

**Methods:**

International, longitudinal multivariate regression analysis of cardiovascular disease mortality data from 1960 to 2002

**Results:**

A system-wide banking crisis increases population heart disease mortality rates by 6.4% (95% CI: 2.5% to 10.2%, p < 0.01) in high income countries, after controlling for economic change, macroeconomic instability, and population age and social distribution. The estimated effect is nearly four times as large in low income countries.

**Conclusion:**

Banking crises are a significant determinant of short-term increases in heart disease mortality rates, and may have more severe consequences for developing countries.

## Background

Fear of financial loss drives people to do irrational things. As the runs on Northern Rock banks in England took place, one could not help but wonder how people's trust in the financial system could have eroded so rapidly.^1 ^Much worse, the spread of panic, in part propelled by media, appears to have turned what could have otherwise been a momentary blip on the financial scene into an economic policy debacle – ultimately leading to a reluctant intervention by the Bank of England and an historic guarantee by the chancellor of the exchequer of *all *Northern Rock deposits in the UK banking system. But the financial storm has not yet passed.^2^

What might be the health implications if the Northern Rock episode develops further into a full-fledged banking crisis in England? To our knowledge, no study has evaluated the relationship between a banking crisis and mortality, even though such crises have occurred more than once every two years in developed countries in the past 30 years. As the current experiences suggest, banking crises impose considerable panic and stress on people and, in particular, on vulnerable older populations. Such acute mental distress has been shown to i) significantly raise heart rate and blood pressure, which may increase myocardial oxygen demand and disrupt vulnerable plaques, and ii) in atherosclerotic patients lead to primary reductions in myocardial oxygen supply via impaired dilatation and vasoconstriction [1-4]. Clinical and experimental studies have documented that extremely stressful events, such as earthquakes [5], wars [6] or terrorist attacks [7,8] are associated with increased risk of acute myocardial infarction and sudden cardiac death.

In the context of a bank system crisis, elderly persons are much more likely to feel threatened by risks to their accumulated savings, and, not surprisingly, the majority of persons who stood in the queue outside Northern Rock appeared to be disproportionately older. Older populations are also the most sensitive to acute financial stress and more likely to have predisposing cardiovascular risk factors such hypertension and hypercholesterolemia. As a result, an acute stressor such as a banking crisis might be expected to raise their short-term risk of fatal cardiac events [5-8].

In this article, we empirically test whether banking crises are linked to increases in cardiovascular mortality rates, using longitudinal data from 1960 to 2002 for high- and low-income countries.

## Methods

Data are drawn primarily from two sources: male cardiovascular mortality rates per 100,000 population from the World Health Organization Global Mortality Database, and years of bank system crises from the World Bank. A bank crisis is defined as an episode in which a significant proportion of banks fail or their assets are exhausted [9]. Since bank crises often last for multiple years, indicators are used for the first year of a country's banking system crisis in order to isolate the short-term effect on mortality.

A set of controls adjusts for potential confounders and surveillance variations. First, as seen in the case of Northern Rock, there is frequently an economic boom prior to a bank system crisis, which may lead to artificially higher or lower mortality rates [10]; hence, models correct for the previous year's change in real gross domestic product per capita. Second, periods of heightened economic uncertainty may increase mortality rates irrespective of whether a banking crisis occurred. Period effects such as these are controlled for by including dummy variables for each year. Third, countries may differ with regard to their surveillance or monitoring of heart disease mortality. A set of dummies for each country are used so that the models evaluate the mortality changes within individual countries while holding constant time-invariant differences between countries including higher predispositions to heart conditions as well as political, cultural and structural differences. In effect, this conservative modeling approach makes the data more comparable. Lastly, controls are used for the population age- and social-distribution (population dependency ratio and urbanization) as well as other measures of macroeconomic flux (log inflation rates) and social development (population average years of education).

Thus we model heart disease mortality rates as follows:

*Log Heart Disease*_*it *_= *α *+ *β*_1_*BANK*_*it *_+ *β*_2_*GDP*_*it*-1 _+ *β*_3_*INFL*_*it *_+ *β*_4_*URBAN*_*it *_+ *β*_5_*DEP*_*it *_+ *β*_6_*EDUC*_*it *_+ *μ*_*i *_+ *η*_*t *_+ *ε*_*it*_

Here *i *is country and *t *is year. Heart disease rates are logged to adjust for positive skew. BANK is the measure of whether a country experienced a banking crisis in the current year; GDP is the previous year's percentage change in real gross domestic product per capita; INFL is the log of inflation in consumer price index; URBAN is the percentage of the population living in urban settings; DEP is the ratio of the youth and elderly to the overall population; EDUC is the average population years of education received; *μ *and η are sets of dummy variables which control for country- and period-specific effects. In order to better extend results to the current United Kingdom crisis, separate models are used for high- and low-income countries, defined as per capita GDP above $25,000 US and less than $2,000 US.

## Results

Table 1 presents the results of longitudinal multivariate regression models of the associated between banking crises and male heart disease mortality in high-income countries from 1960 to 2002. A banking crisis on average is connected with a 6.4% short-term increase in cardiovascular disease mortality (95% CI: 2.5% to 10.1%, p < 0.01) in high income countries, after correcting for prior economic change, inflation levels, population education levels, urbanization, and dependency ratios as well as period- and country-effects. For low-income countries, the estimated effect is roughly four times as large, with a banking crisis corresponding to a 26.0% increase in mortality (95% CI: 2.3% to 49.7%, p < 0.05). However, the sample size diminishes considerably due to the lack of available comparative heart disease data and as a result the confidence intervals are broad enough to where the effect size cannot be distinguished from that in high income countries.

**Table 1:** Effect of a Banking Crisis on Log Heart Disease Mortality Rates by Income Level, 1960–2002

Covariate	High Income Countries	Low Income Countries
Bank Crisis	0.06** (0.02)	0.26* (0.10)
Lag of GDP per capita change	-0.00 (0.00)	0.01* (0.01)
Log Inflation Rate	-0.04** (0.02)	0.10 (0.10)
Urbanization	0.00 (0.01)	0.00 (0.01)
Education Level	0.03 (0.02)	0.14 (0.09)
Dependency Ratio	0.01 (0.00)	-0.00 (0.01)
Number of Observations	729	157
Number of Countries	19	9
R^2^	0.71	0.61

*Note: *Constant estimated but not reported; Robust standard errors in parentheses, clustered by country because observations are not independent. Models include dummy variables for each country and year. High Income countries include Australia, Austria, Belgium, Canada, Denmark, Finland, France, Germany, Japan, Iceland, Italy, Netherlands, New Zealand, Norway, Spain, Sweden, Switzerland, United Kingdom and United States. Banking crisis is defined as a the first year of a systemic banking crisis in which all or most of a country's banking capital is used.^1 ^Urbanization is percentage of population living in urban settings, Dependency ratio is number of elderly and infants as a percentage of total population, Education level is the population average total years of schooling, and the Inflation Rate is based on the change in the consumer price index. R^2 ^value based on within-country variation. Data Sources: World Bank World Development Indicators 2005 edition, World Bank Systemic Banking Crises Data, and World Health Organization Global Mortality Database.

* – p < 0.05, ** – p < 0.01 (two-tailed tests).

How many deaths does the estimated effect correspond to in the United Kingdom? In 2004/2005, there were 50,544 male deaths due to heart disease in the United Kingdom – among the highest rates in OECD countries [11]. If a severe banking crisis were to hit, our results suggest that it would cause anywhere from 1280 to 5130 additional heart disease deaths [3]. To put this effect in perspective, this is more than ten-times the number of British troops who have died in Iraq.

## Discussion

Our results show that bank system crises are associated with short-term increases in heart disease mortality rates, and suggest that this effect may be significantly more pronounced in low-income countries where they occur more frequently. These empirical findings also provide a textbook illustration of how financial globalization matters for health: as a result of US mortgage defaults, Britain's banking sector – and the health of its population – face risks.

Despite the robustness of our findings, there are several important limitations to our analysis. First, as with all cross-national analyses, the potential exists for ecological fallacies. However, the observed associations are biologically plausible, given the established mechanisms by which acute psychological stress increases myocardial ischemia [1-5]. Second, although we control for differences in surveillance between countries, there is potential for bias arising from time-varying surveillance changes within countries. It is, however, unlikely that the temporal variation in surveillance can account for the relationship between banking crises and heart disease net of our control variables, and further the direction of the potential bias is unclear. Third, without more refined data, the epidemiology behind our findings cannot be fully resolved. Even so, the results are almost certainly driven by acute cardiac events which are more likely to have been incident in older population groups. Such non-differential measurement error in our data would have the effect of diluting the regression results, and thus renders our estimates conservative.

Containing the spread of financial hysteria is desirable not only for preventing a systemic bank crisis from occurring but also for avoiding excess cardiac mortality. This study also further supports the availability of cardiac care during stressful episodes such as bank runs when large groups of at-risk individuals experience acute mental distress.

## Conclusion

Northern Rock reminds us that macroeconomic stability is not just about financial health. Whatever one might think of the Bank of England's U-turn, it probably has spared the United Kingdom from a full-scale bank crisis that would have been borne out not only in economic terms but quite possibly in human lives. The governor of the Bank of England, Mervyn King, despite losing some of his tough love reputation, may have helped contribute to a healthier population. The concern remains, however, that by effectively bailing out financial miscreants, the Bank of England may encourage more risky financial behavior in the future (so-called "moral hazard"), and as result increase the risk of a future bank crisis and its associated threats to cardiovascular health.

## Competing interests

The author(s) declare that they have no competing interests.

## Authors' contributions

DS conducted the empirical analysis and drafted the manuscript; CM provided details on the banking crisis, conducted qualitative research during the Northern Rock bank run, and participated in the empirical analysis; LK offered helpful comments and criticisms of various drafts and reviewed the empirical analysis.

## Appendix 1. Endnotes

**1. **In mid-August 2007, the United Kingdom became embroiled in the global financial turmoil that had already hit the US, Germany and France in the early summer of 2007. One of the UK's banks, Northern Rock, had invested in a business plan to borrow heavily in the UK and international money markets, to extend mortgages based on this funding, and then to resell these mortgages on international capital markets. When the global demand dropped in August 2007, Northern Rock became vulnerable to a shutdown in funds. Panic on the financial markets led to further panic among individual depositors that their savings might not be available should Northern Rock go into receivership. This led to a classic bank run – the UK's first in 150 years – where depositors line up outside the bank to withdraw all of their savings as quickly as possible, particularly since everyone else was doing the same. While action by depositors in such a moment was not obviously collectively rational, it was most certainly individually rational.

**2. **See for example "UK still vulnerable to credit squeeze." *Financial Times*, or "Bank of England fears re-run of credit crisis." *The Guardian*, on October 25^th ^2007.
